# Analysis of Prototype Foamy Virus particle-host cell interaction with autofluorescent retroviral particles

**DOI:** 10.1186/1742-4690-7-45

**Published:** 2010-05-17

**Authors:** Kristin Stirnnagel, Daniel Lüftenegger, Annett Stange, Anka Swiersy, Erik Müllers, Juliane Reh, Nicole Stanke, Arend Große, Salvatore Chiantia, Heiko Keller, Petra Schwille, Helmut Hanenberg, Hanswalter Zentgraf, Dirk Lindemann

**Affiliations:** 1 Institut für Virologie, Medizinische Fakultät "Carl Gustav Carus", Technische Universität Dresden, Dresden, Germany; 2 Biophysics, BIOTEC, Technische Universität Dresden, Dresden, Germany; 3 Department of Pediatric Oncology, Hematology & Clinical Immunology, Children's Hospital, Heinrich Heine University, Düsseldorf, Germany; 4 Angewandte Tumorvirologie, Deutsches Krebsforschungszentrum, Heidelberg, Germany; 5 Current Address: ViroLogik GmbH, Henkestr. 91, 91052 Erlangen, Germany

## Abstract

**Background:**

The foamy virus (FV) replication cycle displays several unique features, which set them apart from orthoretroviruses. First, like other B/D type orthoretroviruses, FV capsids preassemble at the centrosome, but more similar to hepadnaviruses, FV budding is strictly dependent on cognate viral glycoprotein coexpression. Second, the unusually broad host range of FV is thought to be due to use of a very common entry receptor present on host cell plasma membranes, because all cell lines tested in vitro so far are permissive.

**Results:**

In order to take advantage of modern fluorescent microscopy techniques to study FV replication, we have created FV Gag proteins bearing a variety of protein tags and evaluated these for their ability to support various steps of FV replication. Addition of even small N-terminal HA-tags to FV Gag severely impaired FV particle release. For example, release was completely abrogated by an N-terminal autofluorescent protein (AFP) fusion, despite apparently normal intracellular capsid assembly. In contrast, C-terminal Gag-tags had only minor effects on particle assembly, egress and particle morphogenesis. The infectivity of C-terminal capsid-tagged FV vector particles was reduced up to 100-fold in comparison to wild type; however, infectivity was rescued by coexpression of wild type Gag and assembly of mixed particles. Specific dose-dependent binding of fluorescent FV particles to target cells was demonstrated in an Env-dependent manner, but not binding to target cell-extracted- or synthetic- lipids. Screening of target cells of various origins resulted in the identification of two cell lines, a human erythroid precursor- and a zebrafish- cell line, resistant to FV Env-mediated FV- and HIV-vector transduction.

**Conclusions:**

We have established functional, autofluorescent foamy viral particles as a valuable new tool to study FV - host cell interactions using modern fluorescent imaging techniques. Furthermore, we succeeded for the first time in identifying two cell lines resistant to Prototype Foamy Virus Env-mediated gene transfer. Interestingly, both cell lines still displayed FV Env-dependent attachment of fluorescent retroviral particles, implying a post-binding block potentially due to lack of putative FV entry cofactors. These cell lines might ultimately lead to the identification of the currently unknown ubiquitous cellular entry receptor(s) of FVs.

## Background

Spumaviruses, also known as foamy viruses (FVs), represent the only genus of the retroviral subfamily spumaretrovirinae, and resemble complex retroviruses with respect to their genome structure. The FV replication strategy deviates in many aspects from that of orthoretroviruses [reviewed in [1]]. Interestingly, many of the unique features of FVs are more reminiscent of another family of reverse transcribing viruses, the hepadnaviridae [reviewed in [2]]. This includes the expression of Pol as a separate protein, instead of the Gag-Pol fusion proteins typical of orthoretroviruses [reviewed in [3]]. As a consequence, FVs have a specific strategy to ensure Pol particle incorporation, essential for generation of infectious virions. Both Gag and Pol proteins of FVs bind to full-length genomic viral transcripts. Additionally, protein-protein interactions between Gag and Pol seem to be involved in this assembly process [4-6]. Other aspects of FV assembly are also unique among retroviruses; for example, while FV Gag can preassemble by itself into capsid structures at the cellular microtubule-organizing-center (MTOC) like B/D type orthoretroviruses, it apparently lacks membrane-targeting signals. Therefore, such particles are not released from the cell as virus-like-particles as observed for other retroviruses [reviewed in [3]]. Similar to Hepatitis B virus (HBV), FV particle budding and release are instead dependent on co-expression of the cognate viral envelope (Env) protein; moreover, this function of FV Env that cannot be complemented by expression of heterologous viral glycoproteins [reviewed in [7]]. A specific interaction between the cytoplasmic N-terminus of the FV Env glycoprotein, involving the leader peptide (LP) and a conserved W_10_XXW_13 _motif, and the N-terminal region of the FV Gag protein, is essential for particle egress. FV Env-independent capsid release can be achieved experimentally by artificial N-terminal fusion of heterologous membrane-targeting signals to the FV Gag. However, these VLPs are non-infectious even when co-expressed with the cognate viral glycoprotein [8-10]. Finally, the structural organization of the FV Gag protein deviates significantly from orthoretroviruses. Unlike orthoretroviral Gag proteins, FV Gag is not processed into separate matrix (MA), capsid (CA) and nucleocapsid (NC) subunits. In fact, only a limited proteolysis is observed during FV particle morphogenesis, resulting in the removal of a C-terminal 3 kD peptide. Both the uncleaved precursor p71^Gag ^and the larger p68^Gag ^cleavage product are incorporated into the FV capsid, where they are found in ratios of 1:1 to 1:4 in released infectious viral particles [11]. Although the FV Gag protein harbors many functional motifs described for other retroviruses (such as an PSAP late assembly (L)-domain, a cytoplasmic targeting and retention signal (CTRS) to mediate assembly at the MTOC, a coiled-coil domain essential for assembly, and a YXXLDL motive important for capsid morphology and reverse transcription), other motifs are either missing from FV Gag or if present, are unique amongst retroviruses [8,12-15]. This includes the lack of C-terminal Cys-His boxes in Gag implicated in retroviral RNA packaging [reviewed in [3]]. Instead up to three glycine-arginine-rich sequences (GR-boxes) are found in the C-terminal region of FV Gag. GR-I was reported to bind to nucleic acids and was originally implicated in RNA binding, but this was recently challenged and another function as an interaction motif for the Gag-Pol interaction during Pol particle incorporation was described [4,16]. GR-II harbors a nuclear localization signal sequence responsible for predominant nuclear targeting of FV Gag at certain time points during viral replication [16,17]. Furthermore, recently a chromatin-binding site (CBS) within GR-II was identified mediating attachment of FV Gag to host chromosomes [18].

In recent years, the combination of fluorescently labeled virions with modern imaging techniques has proven to be a powerful tool to study replication in a variety of viral systems. These methods have been particularly useful for dissecting assembly and entry pathways [reviewed in [19]]. With respect to retroviruses, single virus tracking has revealed that Murine Leukemia Virus (MLV) infection induces establishment of filopodial bridges that enable efficient cell-to-cell transmission; has allowed the quantitation of individual HIV particle genesis in real time; and enabled detailed analysis of the very earliest events during HIV attachment to target cells [20-22].

Further analysis of the FV replication strategy would profit greatly from the availability of functional fluorescent FV particles. For example, the exact cellular location of FV Gag - Env interaction could be determined and examined by time-lapse microscopy. Originally it was thought to occur at the membrane of the endoplasmic reticulum, since FV Env contains an ER retrieval signal and budding seemed to occur at intracellular membranes, which are believed to be the ER. However, Yu et al. reported recently a significant Gag - Env co-localization only in compartments containing Golgi-specific marker proteins, in a study using FV infected fibroblasts and immunostaining of fixed samples [23]. Similarly, the cellular location of the Gag - Pol interaction is currently unknown, and its identification would contribute to the understanding of FV Pol particle incorporation mechanism. Furthermore, very little is known about the sequential events leading to FV entry of target cells, and live imaging of FV uptake could lead to insights into the entry mechanism of these unusual retroviruses.

Currently, it is thought that FV particles bind to a ubiquitous, but as yet unidentified, cellular receptor. This is based largely on the observation that FVs are unique amongst retroviruses in having an extremely broad host range [24,25]. FV vectors can transduce even bird or reptile cells. Indeed, a species or cell type that is completely resistant to FV Env-mediated transduction has not been reported. After attachment, FV capsids apparently are endocytosed, gaining access to the cytoplasm by a FV Env-mediated pH-dependent fusion process, and seem to migrate to the centrosome by piggybacking on dynein/dynactin motor complexes [26,27]. There they can reside for long periods of time until disassembling and progressing towards nuclear entry of the FV preintegration complex, induced by yet uncharacterized cellular signals [28].

A few previous studies have employed enhanced green fluorescent protein (EGFP) tagged FV Gag proteins for cellular assays [9,18,26]. Petit et al. [26] and Tobaly-Tapiero et al. [18] used different, transiently-expressed N-terminal tagged Gag proteins to characterize the centrosome-targeting and chromatin-binding motifs in PFV Gag. The influence of L-domain mediated Gag ubiquitination on retroviral budding was examined by Zhadina et al. [9] using artificially membrane-targeted, Env-independently budding PFV Gag protein containing a C-terminal GFP-tag. However, the functional consequences of tagging the FV Gag proteins, compared to untagged wild type FV Gag protein, were not examined in these studies.

In this study, we systematically analyzed the influence of different protein tags on PFV Gag's capacity to support FV replication using recombinant replication-deficient FV vector particles that are capable of single-round infections. We succeeded in identifying for the first time autofluorescent protein (AFP)-tagged PFV Gag constructs that allow generation of fluorescent PFV particles with nearly wild type functionality; these constructs provide a powerful tool for analysis of PFV replication steps by modern imaging techniques. With this tool, a particle-binding assay for target cells was established. In combination with high-titer FV Env containing retroviral vector supernatants, it was used to identify two cell lines that are resistant to PFV Env-mediated marker gene transfer. Interestingly, these cells still displayed retroviral particle attachment in a FV Env-specific manner. Further characterization of the resistance to FV Env-mediated virus entry in these cell lines might ultimately lead to the discovery of currently unknown cellular molecules essential for the early stages of FV infection in target cells.

## Results

### Peptide length and location influence function of tagged PFV Gag

**Figure 1:**
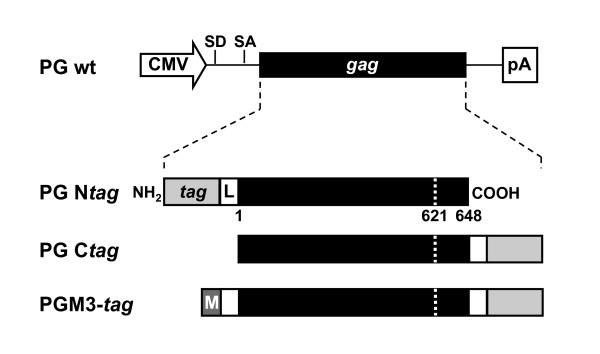
**Schematic illustration of the PFV Gag (PG) fusion expression constructs**. CMV, cytomegalovirus virus promoter; SD, splice donor; SA, splice acceptor; pA, bovine growth hormone polyadenylation signal; L, glycine-serine linker. The p68/p71 PFV Gag cleavage site is shown as dashed line. PFV Gag fusion proteins were generated as N- or C-terminal fusions. The locations of the different protein tags (HA, eGFP, eYFP, mCherry, mCerulean) used are indicated as grey boxes (*tag*). The C-terminal PG CeGFP fusion protein was further modified by N-terminal fusion of a membrane-targeting signal (M) (PGM3).

**Figure 2:**
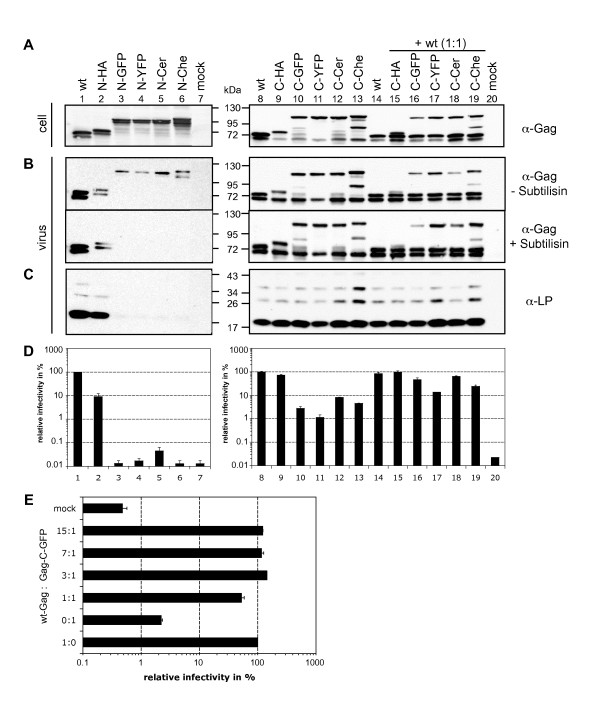
**Cellular and particle associated protein expression- and infectivity analysis of PFV Gag-FPs**. PFV particles were generated by transient transfection of 293T cells using the 4-plasmid PFV vector system. (A-C) Representative Western Blot analysis of 293T cell lysates (cell) (A) and viral particles (virus) purified by ultracentrifugation through 20% sucrose for N- or C-terminal Gag-FPs (B, C). PFV proteins were detected by using (A, B) a polyclonal anti-PFV Gag (α-Gag) or (C) an anti-PFV Env LP (α-LP) specific antiserum. (B) In addition subtilisin- and mock-treated samples were compared according to their particle associated Gag expression by α-Gag immunoblot. (D) Relative infectivities of extracellular cell culture supernatants using EGFP marker gene transfer assay. The values obtained using wild-type PFV Gag expression plasmids (lane 1, 8) were arbitrarily set to 100%. Mean values and standard deviations from three independent experiments are shown. 293T cells were cotransfected with puc2MD9, pcziPol, pczHFVenv EM002 and either (lane 1, 8) pcoPG4 (wt), (lane 2) pcoPG4 NHA, (lane 3) pcoPG4 NeGFP, (lane 4) pcoPG4 NeYFP, (lane 5) pcoPG4 NCerulean, (lane 6) pcoPG4 NmCherry, (lane 9) pcoPG4 CHA, (lane 10) pcoPG4 CeGFP, (lane 11) pcoPG4 CeYFP, (lane 12) pcoPG4 CCerulean, (lane 13) pcoPG4 CmCherry or wtGag cotransfected at a ratio of 1:1 (lane 14) pcDNA 3.1zeo+, (lane 15) pcoPG4 CHA, (lane 16) pcoPG4 CeGFP, (lane 17) pcoPG4 CeYFP, (lane 18) pcoPG4 CCerulean, (lane 19) pcoPG4 CmCherry. As control, cells were only transfected with pcDNA3.1 zeo+ (lane 7, 20). (E) Comparison of relative infectivities of C-terminal Gag-GFP (Gag-C-GFP) fusion proteins either transfected alone or cotransfected with untagged Gag (wt-Gag) with the EGFP marker gene transfer assay, as depicted. The values obtained using wild-type PFV Gag expression plasmids (1:0) were arbitrarily set to 100%. Mean values and standard deviations from two independent experiments are shown. 293T cells were cotransfected with puc2MD9, pcziPol, pczHFVenv EM002, pcoPG4 (wt) or/and pcoPG4 CeGFP at different ratios as indicated.

We set out to establish a collection of tagged PFV Gag proteins that retain most of their natural functions essential for FV replication. With these tools we aim to study various steps of the FV replication strategy in host cells by combining different biochemical assays with modern live-cell imaging techniques. Towards this end we generated expression constructs containing different protein tags fused in frame with the PFV Gag ORF (Fig. 1). Recombinant PFV vector particles containing these Gag fusion proteins (Gag-FPs) were produced by transient transfection of 293T cells using a 4-plasmid PFV vector system [29]. Subsequently, cellular protein expression, particle-associated protein composition, and infectivity of recombinant vector particles were examined. Biochemical analysis of cell lysates revealed that all Gag-FPs were expressed and processed at levels slightly lower or similar to untagged PFV Gag (Fig. 2A). Increases in the observed molecular weight of the individual tagged Gag proteins were consistent with the predicted size of the different peptide tags added. For N-terminal tagged Gag proteins, both the p71^Gag ^and p68^Gag ^displayed a higher mass in comparison to untagged PFV Gag (Fig. 2A, lane 1-6). In contrast, for the C-terminal tagged Gag proteins, only the p71^Gag ^precursor protein showed a higher molecular weight because normal C-terminal proteolytic processing led to authentic p68^Gag ^cleavage products lacking the tag (Fig. 2A, lane 8-13). Initial analysis of particle release, by particle concentration through ultracentrifugation and subsequent Western blot analysis using FV specific antisera, revealed that all of the tagged PFV Gag proteins appeared to support particle egress (Fig. 2B). However, in general, the release of capsid containing N-terminal tagged Gag proteins was significantly decreased in comparison to wild type (Fig. 2B, lane 1-6). Furthermore, in the lysates of the larger N-terminal AFP-tagged Gag protein particle preparations no viral glycoprotein was detectable, evidenced by the lack of PFV Env LP specific signals (Fig. 2C, lane 3-6). In contrast, particle lysates of the smaller N-terminal HA-tagged Gag displayed incorporation of the PFV Env LP subunit (Fig. 2C, lane 2).

To investigate whether detected Gag proteins were particle-associated or extracellular protein aggregates, purified particle samples were digested with the membrane-impermeable protease subtilisin, prior to particle lysis (Fig. 2B; lower panel). By this treatment, all viral protein components not enveloped and protected by a lipid membrane are removed. Indeed, we observed that in all N-terminal Gag-AFP samples the Gag-specific signals detected in duplicates that were mock treated (Fig. 2B, lane 3-6, upper panel) disappeared upon subtilisin digestion (Fig. 2B, lane 3-6; lower panel). All other samples, including N-terminal HA-tagged- and all C-terminal tagged Gag proteins, were unaffected by proteolytic digestion and appear as Gag-specific signals in the Western Blot analysis (Fig. 2B, lanes 1, 2, 7-20; compare upper and lower panel). Remarkably, there was an additional prominent protein band in all C-terminal tagged mCherry-Gag samples, recognized with both Gag-specific and mCherry-specific antibodies (Fig. 2A, B, lane 13, 19; data not shown). This protein most probably is the result of an internal mCherry cleavage, which has been described in the literature, and is thought to be involved in maintaining the functional chromophore of this fluorescent protein [30-32].

We further observed that the small HA-tag fused to the N-terminus of Gag significantly reduced particle release efficiency in comparison to wild type, which was not observed for the C-terminal HA-tagged Gag-FP (Fig. 2B, lane 1, 2, 8, 9). These effects of HA-tag addition on particle release were in accordance with the calculated relative infectivities depicted in Fig. 2D. Samples of N-terminal HA-tagged particles showed a 10-fold reduction of supernatant-associated infectivity, whereas those of C-terminal HA-Gag-FP particles were almost at wild type levels (Fig. 2D, bar 1, 2, 8, 9). This suggests that the PFV Gag N-terminus is more sensitive to modifications than the C-terminus. Furthermore, addition of different AFPs to the N-terminus of Gag almost completely abolished release of infectious particles (Fig. 2D, bar 3-6). This observation is in line with the inability of these proteins to support release of lipid membrane enveloped Gag protein (Fig. 2B, lane 3-6). In contrast, the range of supernatant infectivity measured for C-terminal Gag-AFPs was between 1 - 8% compared to untagged wild type samples (Fig. 2D, bar 8, 10-13). Since the physical particle release of these samples was almost equal to wild type (Fig. 2B, lane 8-13), this reduction in measurable infectivity indicates that a larger C-terminal fusion tag might interfere with replication steps other than particle release. No major difference in the relative incorporation and processing of Pol was observed in released particles of the individual Gag mutants (data not shown). To examine if untagged wild type PFV Gag protein is able to rescue the particle release and infectivity defects observed for some of the Gag-FP, we cotransfected expression constructs of both type of proteins at various ratios (Fig. 2; and data not shown). In Fig. 2E, the influence of cotransfection of various ratios of wild type Gag with C-terminal tagged Gag-GFP on supernatant infectivity is shown. By increasing the ratio of wild type Gag protein to tagged protein the infectivity could be restored, reaching wild type levels at a 3:1 ratio of wild type to tagged Gag protein and 50% infectivity levels at a 1:1 ratio. For the N-terminal tagged Gag-GFP, cotransfection of wild type Gag was unable to restore supernatant infectivity to wild type levels, even at a 15-fold excess of wild type Gag expression construct (data not shown). This suggests a dominant negative effect of the N-terminal Gag-GFP fusion. Subsequently, physical particle release of all fusion proteins was analyzed at a 1:1 cotransfection ratio and compared to conditions without wild type Gag protein coexpression (Fig. 2A-D; and data not shown). For all C-terminal tagged Gag constructs a similar ratio of tagged and wild type protein was detected in corresponding cell and particle lysates (Fig. 2B, lane 14-20). In contrast, no tagged Gag protein was observed in particle lysates of samples cotransfected with N-terminal AFP-tagged constructs (data not shown). Supernatant infectivities of the C-terminal tagged constructs were restored to 15-100% of wild type levels independent of the specific tag sequence used. The relative differences in infectivities between the various tagged constructs were similar, independent of wild type Gag protein coexpression. Thus, C-terminal, but not N-terminal AFP-tagged PFV Gag proteins, can interact with wild type Gag protein to allow release of mixed particles with greatly improved specific particle infectivity.

### C-terminally tagged Gag-AFPs display nearly normal capsid structures and budding characteristics

**Figure 3:**
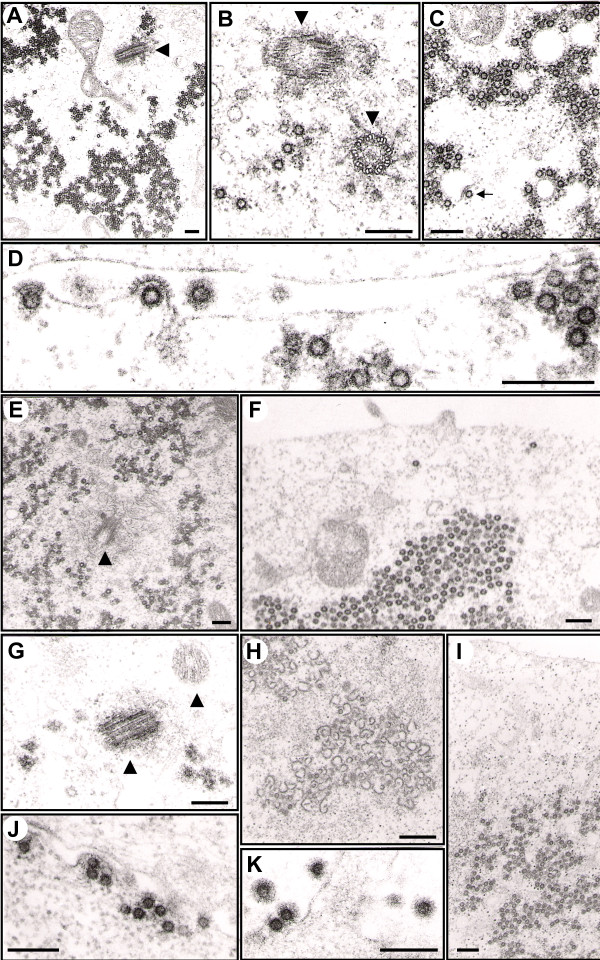
**Electron microscopy analysis of transfected 293T cells**. Electron micrographs showing representative thin sections of transiently transfected 239T cells using the 4-plasmid vector system. (A-D) Untagged PFV Gag expression construct. Arrowheads point to centrioles (MTOC, microtubule organizing center). The arrowhead points to a budding particle into intracellular vesicles. (E-F) N-terminal Gag-GFP expression construct. (G-K) C-terminal Gag-GFP expression construct. Magnifications: (A) 18000×, (B) 58000×, (C) 41000×, (D) 117000×, (E) 23000×, (F) 33000×, (G) 47000×, (H) 20000×, (I) 28000×, (J) 65000×, (K) 71000×. scale bar: 200 nm.

Due to the apparently decreased infectious titer of several Gag-AFP tagged particles observed, we were interested in taking a closer look at the particle morphology of these fluorescent viruses. Therefore, we used ultrastructural EM (electron microscopy) to analyze 293T cells expressing different GFP-tagged Gag-FPs in the context of the 4-plasmid FV vector system (Fig. 3).

Wild type unmodified Gag proteins were found to assemble into homogenous spherical capsids accumulating intracellularly in large amounts mainly at the MTOC (microtubule organizing center), as previously reported (Fig. 3A, B). Furthermore, particle budding was observed into intracellular vesicles and to a large extent also at the plasma membrane, sometimes associated with capsids aggregating at the plasma membrane (Fig. 3C, D). Similar to wild type PFV Gag, N-terminal tagged Gag-GFP also assembled into capsids with wild type morphology and accumulating mainly at the MTOC (Fig. 3E, F). However, in these samples no budding profiles could be detected (Fig. 3E, F; and data not shown). This is in line with the biochemical analysis (Fig. 2B, C) and indicate that the lack of particle release may be due to a failure of the N-terminal tagged Gag-AFP to successfully interact with PFV Env, an interaction that is essential for capsid-membrane association. In contrast, C-terminal Gag-GFP-FPs were found to bud at the plasma membrane, indicating that a functional Gag-Env interaction occurs and that the GFP tag does not influence late budding events (Fig. 3J, K). In this case, capsid morphology seemed to be slightly more heterogeneous compared to untagged capsids. But capsids were also found to accumulate at the MTOC, and budding structures containing the typical prominent FV Env spike structures at the plasma membrane were observed (Fig. 3G, I, J, K). Remarkably, in some cells in these samples, we detected intracellular accumulation of potentially aberrant capsid structures which might represent sites of protein degradation (Fig. 3H). These curious structures were neither found at the budding site nor in released viruses of C-terminal tagged PFV Gag samples nor in samples of other tagged or wild type Gag constructs. This suggests that C-terminal AFP tags to the PFV Gag protein may result in some minor interference with intracellular capsid assembly, however, all budding and released virions displayed wild type morphology.

### EYFP and EGFP are the most convenient tags to analyze PFV capsids by fluorescence microscope techniques

**Figure 4:**
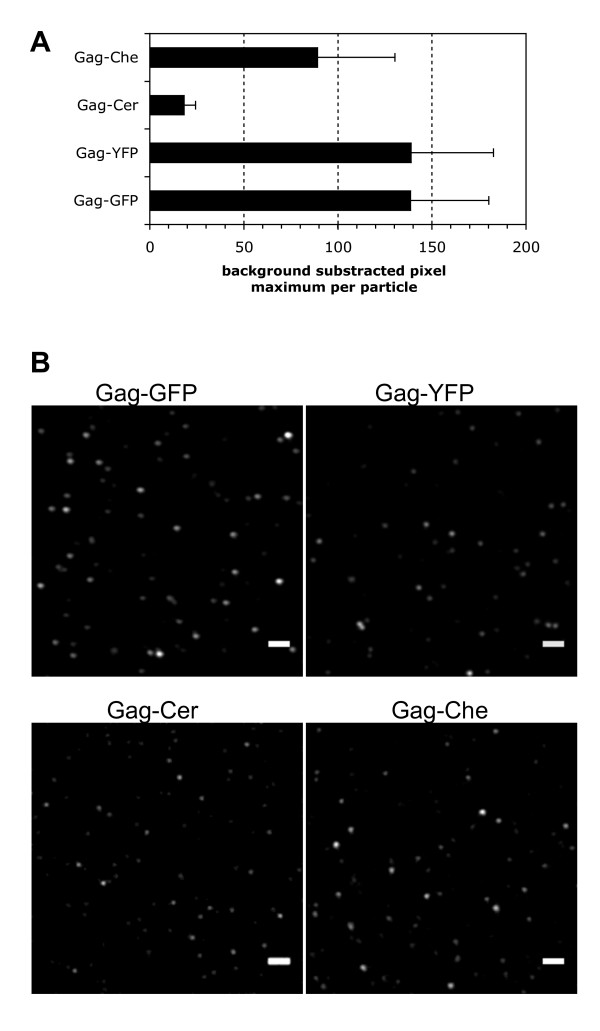
**CLSM analysis of purified PFV Gag-labelled particles**. Viruses were produced by transfecting 293T cells with expression plasmids for Env, Pol, RNA and the appropriate C-terminal tagged Gag-AFP and harvested by ultracentrifugation. Subsequently purified virus was incubated on glass cover slips, fixed and the samples covered in Mowiol. (A) Comparison of fluorescence intensities of background subtracted and smoothed pictures (ImageJ plugins). The mean of at least three randomly taken areas of each particle population was determined. Average and Standard Deviation are depicted. (B) Confocal Laser Scanning Microscopy (CLSM) analysis revealed, that only GFP and YFP labelled virus were efficiently detected inside virus capsids. Although all four fluorescent Gag fusion proteins are incorporated into released particles at comparable amounts (compare with Fig. 2), particles made by mCerulean- or mCherry-Gag were only marginally detectable.

Since the biochemical analysis revealed that all four C-terminal tagged autofluorescent Gag-FPs mediate particle release of infectious virions, we were interested to determine if single fluorescent particles can be imaged by Confocal Laser Scanning Microscopy (CLSM). For this purpose particles purified by ultracentrifugation were spotted onto glass cover slips, fixed and further analyzed by CLSM. The results obtained are summarized in Fig. 4. Whereas EGFP and EYFP tagged PFV particles could be detected very easily, mCherry and mCerulean modified virus particles showed very low signal intensities (Fig. 4A). Although mCerulean and mCherry were incorporated into particles (Fig. 2B, lane 12, 13), they were only detectable by making "blind scans". Subsequent image correction with ImageJ plugins and further modifications of brightness and contrast levels, finally led to the images shown in Fig. 4B. The particle signal intensities calculated from non-modified original scan pictures and the results given as average of the maximum pixel values per particle (n = 30) are shown in Fig. 4A. Furthermore, no GFP signals were detected in mock-purified supernatants of 293T cells, which were cotransfected with pcoPG4 CeGFP in the context of the 4-plasmid vector system lacking an Env expression plasmid (data not shown). Thus PFV Gag-AFP proteins seem to be released in particulate forms in a PFV Env-dependent manner, like the wild type protein.

### Gag-GFP labelled PFV particle preparations contain single viruses

**Figure 5:**
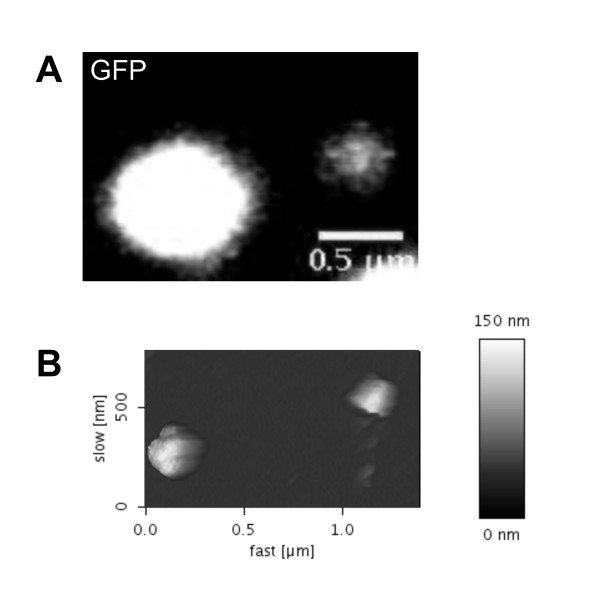
**Comparative analysis of Gag-GFP labelled PFV particles by CLSM and AFM**. Panel A shows the CLSM image of a 100 nm fluorescent bead (on the left) and a PFV virion (on the right) supported on poly-D-lysine coated mica. The high PMT electronic gain necessary to detect the signal from the PFV virion resulted in saturation of the pixels corresponding to the fluorescent bead. Panel B shows the topographical AFM image of the same part of the sample shown in panel A.

We were interested in verifying that autofluorescent PFV particle preparations contain predominantly single virions and not aggregates. For this purpose a comparative ultrastructural analysis on C-terminal Gag-GFP-tagged PFV particle preparations was applied. Labelled virions were harvested by ultracentrifugation and simultaneously fixed in paraformaldehyde. Purified PFV particles were prepared for a combined AFM (atomic force microscopy) and CLSM analyses, performed as described in materials and methods. They were mixed prior to analysis with fluorescent beads (100 nm in diameter) to obtain topographical landmarks useful for alignment of AFM and CLSM scans resulting in three important advantages. First, the same excitation wavelength (488 nm) could be used for Gag-GFP labelled virions and fluorescent beads. Furthermore, CLSM scans nicely show oversaturated beads located next to less intensive GFP-tagged particles, a typical example of which is shown in Fig. 5A. Second, applying distance measurement analysis between beads and particles in the CLSM scan enabled identification of the appropriate GFP-tagged particles in the AFM scan (Fig. 5B). Third, the bead diameter of 100 nm gave us the possibility to compare the size of PFV particles in the AFM scan. In cross section analysis the average height of single PFV particles was calculated as 85 nm (n = 11, standard deviation 13 nm; data not shown). Thus combined AFM- and CLSM analysis confirmed that C-terminal AFP-tagged PFV particle preparation contained predominantly single virions.

### PFV particles bind to the host cell surface, but not to extracted host cell lipids

One special feature of the FV life cycle is an extremely broad host range. To date, there are no reports identifying species, tissues or cell types that are not susceptible to FV Env-mediated transmission. This suggests that the FV receptor molecule(s) is evolutionarily well conserved and present on most if not all eukaryotic cell membranes. We were interested in using the functional fluorescently-tagged PFV particles described above as a tool to measure and visualize potential virus-receptor interactions.

**Figure 6:**
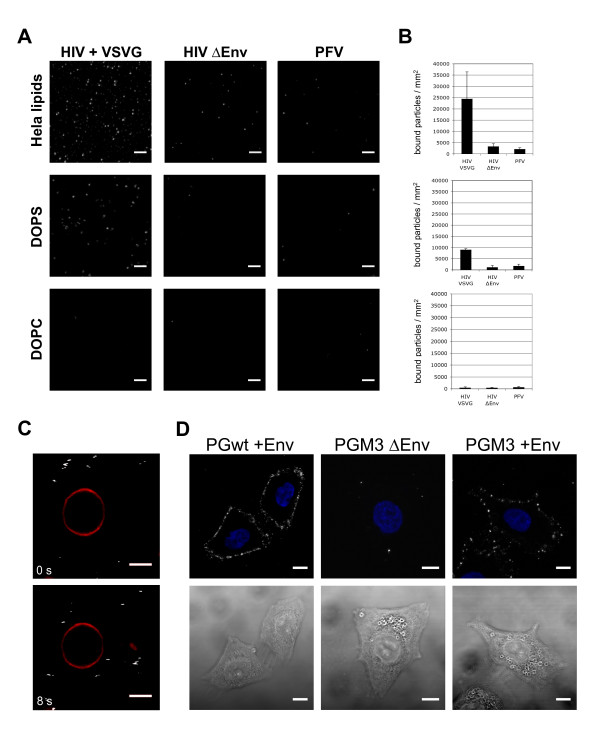
**CLSM analysis of Gag-GFP labelled virus binding to host cell lipids**. (A) Incubation of concentrated PFV, VSV-G pseudotyped HIV particles and HIV VLPs (ΔEnv) with extracted HeLa lipids or synthetic lipids (DOPC/DOPS, DOPC). On DOPC (Dioleoyl phosphatidylcholine), a synthetic neutral phospholipid, none of the particles bound. The mixture containing 30% negatively charged DOPS (Dioleoyl phosphatidylserine), which is necessary to mediate VSV-G particle binding, interacted with HIV VSV-G pseudoparticles. Binding to extracted lipids from HeLa cells (Hela lipids) was only detectable for HIV VSV-G pseudoparticles. Scale bars: 5 μm. (B) The total amount of particles bound to the lipid surface was quantified by automated image analysis (average of 3 scanned areas and 3 scans each). (C) Concentrated Gag-GFP labelled PFV particles (grey channel) were incubated with GUVs (Giant Unilamellar Vesicles, red channel), prepared from HeLa lipids and the a far-red lipid dye DiD-C18. No particle binding to the lipid membrane was observed. Images of the same GUV at two different time points (0s, 8s) are shown. Scale bar: 5 μm. (D) Binding of GFP labelled wt (PGwt) or PGM3 derived (PGM3) PFV particles containing (+Env) or lacking (ΔEnv) PFV Env (grey channel, upper panel) to the cell surface of HeLa cells. Nuclei were stained with DAPI (blue channel). The corresponding DIC images are shown below.

Host cell lipids, in addition to proteins and carbohydrates, are the major constituent of cellular membranes and are also implicated in uptake mediated by VSV-G, a viral glycoprotein displaying a broad host range similar to the FV Env protein [33,34]. The potential involvement of host cell lipids for FV Env mediated entry was tested using two approaches. First, synthetic lipids or a lipid mixture extracted from the FV susceptible human cell line HeLa were spotted onto a glass slide. Subsequently, several differently tagged viral particle preparations, normalized for physical particle concentration, which was determined by FCS, were incubated with the spotted lipids. After extensive washing, particle binding was examined by CLSM (Fig. 6A). GFP-tagged HIV-VSV-G pseudoparticle binding was detectable for HeLa lipids containing phosphatidylserine (PS) and to a slightly lower extent for a mixture containing 30% synthetic PS (DOPS, dioleoyl phosphatidylserine) and 70% DOPC (dioleoyl phosphatidylcholine), but not for DOPC alone (Fig. 6A+B, left column). In contrast, both GFP-tagged HIV virions lacking a viral glycoprotein and GFP-tagged PFV virions displayed minimal or no binding capacity to any of the lipids examined (Fig. 6A+B, center and right column). In a second approach, HeLa cell lipid extracts were used to generate giant unilamellar vesicles (GUV). Control experiments showed that these lipid extracts contained both charged lipids as PS and glycosylated lipids as GM1 (data not shown). But incubation of these GUVs with purified EGFP-tagged PFV virions for up to 30 minutes followed by CLSM analysis of the samples resulted in no indication of FV particle attachment to the GUV surface (Fig. 6C), whereas HIV-VSV-G pseudotype particle binding was clearly detectable (data not shown). Labelled PFV virion signals were only detectable in the liquid surrounding the GUVs (Fig. 6C). Thus, neither lipids extracted from susceptible cells by the method employed nor selected synthetic lipids seem to contribute to PFV particle attachment.

Second, we examined the capacity of fluorescent PFV particles to bind to target cells. For this purpose HeLa cells were incubated with concentrated GFP-tagged PFV virions, followed by extensive washing and subsequent investigation by CLSM analysis. Binding of Gag-GFP-labelled particles to the surface of HeLa cells was readily detectable (Fig. 6D, PGwt +Env). Since particle release of FVs is strictly glycoprotein-dependent, we were unable to assess the binding capacity of FV VLP lacking FV Env. Therefore we made use of a PFV Gag mutant (PGM3) that contains a heterologous N-terminal membrane-targeting signal to examine the FV Env-independent binding capacity of FV virions. Similar PFV Gag proteins were reported previously to enable Env-independent PFV particle release [8,9]. As illustrated in Fig. 6D GFP tagged PGM3 virions harboring PFV Env (PGM3 +Env) were capable of attaching to the HeLa cell surface whereas GFP tagged PGM3 virions generated in the absence of PFV Env coexpression (PGM3 ΔEnv) had a strongly reduced binding capacity. Thus, specific binding of GFP-tagged virus to target cells was observed.

**Figure 7:**
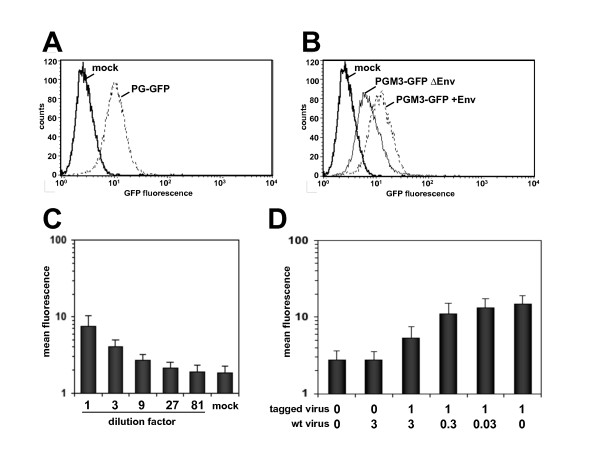
**FACS analysis of PFV particle binding to HeLa cells**. (A, B) Histogram data of measured GFP signal intensities obtained after incubation of (A) GFP-tagged wt (PG-GFP) or (B) PGM3 derived (PGM3-GFP) PFV particles, containing (+Env) or lacking (ΔEnv) PFV Env, with HeLa cells. (C) Target cell attachment of Gag-GFP labelled PFV virions was dose-dependent. (D) GFP-tagged particle binding could be competed for by preincubation with untagged PFV particles. HeLa cells were preincubated with untagged PFV particles at different concentrations. After preincubation with untagged PFV particles, the virus-containing solution was replaced by GFP-tagged viruses at equal amounts in each sample.

Subsequently, a more quantitative and sensitive flow cytometric assay to assess target cell binding of GFP-tagged PFV virions was established. A clear shift in the mean fluorescence intensities was observed upon incubation of HeLa cells with wild type Gag-GFP-labelled particles (PG-GFP) in comparison to mock treated cells (mock) (Fig. 7A). Further this shift was also obtained for PGM3-GFP labelled particles harboring PFV Env (PGM3-GFP +Env) in comparison to those lacking PFV Env (PGM3-GFP ΔEnv) or mock-treated cells (mock) (Fig. 7B). However, a significant binding activity of Env-deficient PGM3-GFP particles (PGM3-GFP ΔEnv) was detected on HeLa cells in comparison to mock-incubated cells (mock), implying an Env-independent component of FV particle attachment to target cells similar to previous reports for other retroviruses [35]. Target cell attachment of Gag-GFP labelled PFV virions was dose-dependent (Fig. 7C) and could be competed for by untagged PFV particles (Fig. 7D).

### Identification of cell lines resistant to PFV-Env mediated vector transduction

**Figure 8:**
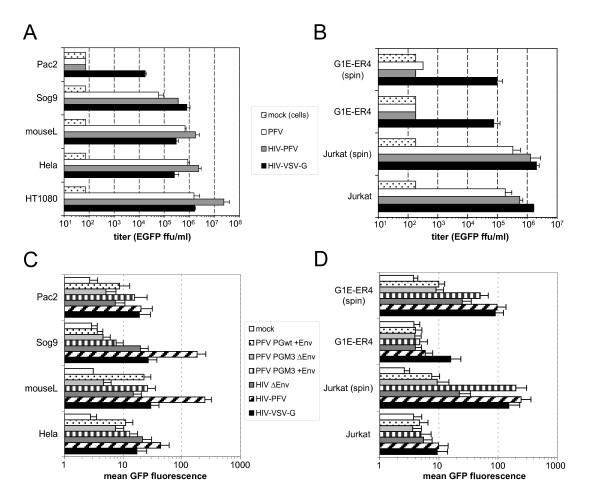
**Host cell characteristics of PFV Env-mediated transduction and Gag-GFP particle binding**. (A, B) Evaluation of different retroviral vector titers on various adherent- (A) or suspension (B) cell lines. mock: non-infected cells; PFV: PFV vector transduced; HIV-PFV: HIV - PFV Env pseudotype transduced; HIV-VSV-G: HIV - VSV-G pseudotype transduced. (C, D) Comparison of mean EGFP fluorescence signals of various adherent- (C) or suspension (D) cell lines after incubation with different Gag-GFP-tagged retroviral particles. Mock: PBS incubated; PFV PGwt +Env: PFV Env containing C-GFP-tagged PFV Gag wt particles; PFV PGM3 ΔEnv: Env-deficient C-GFP-tagged PFV Gag PGM3 particles; PFV PGM3 +Env: Env containing C-GFP-tagged PFV Gag PGM3 particles; HIV ΔEnv: Env-deficient GFP-tagged HIV VLPs; HIV PFV: PFV Env pseudotyped HIV VLPs; HIV-VSV-G: VSV-G pseudotyped HIV VLPs.

Previous attempts to identify cell lines non-permissive for FV infection proved to be unsuccessful [24,25]. We extended the analysis of FV-Env mediated host range further by challenging target cells of various origins with high-titer supernatants of PFV vectors and HIV-1 VSV-G or PFV Env pseudotypes (Fig. 8A, B). First, we examined whether proteoglycans are essential for PFV transduction by comparing the transduction efficiency of mouse L-cell and a proteoglycan synthesis-deficient subclone thereof called Sog9 [36]. As shown in Fig. 8A, Sog9 cells were 2-3 fold better transduced by HIV-1 VSV-G pseudotypes than parental mouse L cells. In contrast, PFV Env-mediated transduction of PFV or HIV-1 PFV Env pseudotypes was diminished about 10-fold on the Sog9 cell line in comparison to parental mouse L cells; nevertheless Sog9 cells were still clearly susceptible to PFV Env-mediated entry. This indicates that proteoglycans are not absolutely essential for FV susceptibility, although they seem to contribute to significant extent to PFV Env-mediated infection efficiency. Second, we examined transduction efficiencies of various other target cells including cells of the human hematopoietic lineage and other species (Fig. 8A, B; and data not shown). All target cell types examined were clearly susceptible to VSV-G mediated marker gene transfer (Fig. 8A, B; and data not shown). However, the extensive analysis led to the identification of two cell lines apparently resistant to PFV Env-mediated vector transduction (Fig. 8A, B). No infectivity of PFV Env containing vector supernatants was detectable on the zebrafish cell line Pac2 (Fig. 8A) and the human erythroid precursor cell line G1E-ER4 (Fig. 8B) even after transduction by spinoculation. In contrast, VSV-G pseudotype titers were 500-fold above the detection limit.

Finally, we examined these two cell lines, along with susceptible adherent and suspension cell lines as controls, for their retroviral particle binding capacity. Therefore the flow cytometric assay using various GFP-tagged retroviral virions as described above was used to get a first idea at which step of the entry process the block might occur (Fig. 8C, D). Most adherent and suspension cell lines examined clearly bound VSV-G containing HIV particles, in line with the transduction data (Fig. 8C, D), although significant cell-type specific differences in relative binding capacities of corresponding Env-less or Env-containing particles were observable. Binding of FV Env containing particles but not of VSV-G pseudotypes was diminished on the proteoglycan synthesis deficient Sog9 cells in comparison to the parental mouse L-cells (Fig. 8C). This is in line with the transduction susceptibility data of these cell lines. Interestingly, Pac2 cells still specifically bound different FV Env containing PFV particles (Fig. 8C). The same was observed for G1E-ER4 cells; however, here clearly specific binding of FV Env-containing virus was only observable by using spinoculation (spin) during particle incubation with the target cells (Fig. 8D). Taken together, these data suggest that both cell lines still demonstrate a FV Env specific attachment of retroviral particles, and a currently unknown post-attachment block might be responsible for the failure of FV Env-mediated marker gene transfer and expression by different retroviral vectors in these target cells.

## Discussion

Addition of peptide tags to proteins can interfere with their natural function, often in a position- and size-dependent fashion. In particular, if no high-resolution structural information on the protein of interest is available, as is the case for the FV Gag protein, a careful functional characterization of the fusion protein in comparison to its wild type counterpart is necessary to allow its further use to address specific scientific questions. Here, we added various tags at different positions of the PFV Gag protein and examined their effects on biological function in comparison to the unmodified wild type protein. In general we observed that a small HA-tag had no or only minor effects in contrast to the more severe consequences of larger AFP tags. Furthermore, fusions to the N-terminus of PFV Gag were more detrimental than C-terminal fusions.

Whereas no major difference in capsid assembly and morphology could be observed for N-terminal tagged PFV Gag proteins by ultrastructural analysis, the small HA-tag significantly decreased particle export; export was reduced even more drastically, to undetectable levels, when the larger AFP tags were used. This is consistent with the current view, that the N-terminal part of FV Gag is involved in a specific interaction with the cognate Env protein that is essential for budding and particle release [10,14,37]. Larger tags may lead to a stronger steric interference than smaller ones. In line with these data, the sensitivity of the FV Gag N-terminus towards modifications has been reported previously for Env-independently budding Gag variants containing N-terminal replacement or additions of heterologous membrane (M) targeting signals [8,9]. When examined these particles were non-infectious even upon FV Env coexpression. Similarly, the PGM3 mutant used in this study was non-infectious as well (data not shown). Furthermore, a detailed mutagenesis and replacement analysis of the N-terminal 11 aa of PFV Gag by Life et al. [10] revealed that M signal replacement abolishes infectivity to undetectable levels and leads to gross morphological defects. Pelletable mutant Gag particles contained little genomic RNA and deviated in density from wild type. Similarly, we observed release of small extracellular amounts of N-terminal AFP-tagged Gag but no measurable infectivity. Although pelleted like viral particles, they were not protected against proteolytic digestion by subtilisin. This strongly suggests for these mutants a release of AFP-Gag aggregates rather than regular VLPs, which is further supported by the nearly undetectable Env subunit levels in these particle lysate samples.

FV capsids have been reported to accumulate near the centrosome initially during virus entry, but later on also upon particle assembly [8,23,26,38], suggesting that microtubule-dependent transport processes are involved for both steps of the FV replication cycle. The study by Petit et al. [26] investigating FV entry processes utilized N-terminal GFP-tagged PFV Gag proteins and identified an interaction with dynein light chain 8 (LC8) as an essential step for MTOC targeting of incoming particles. However, rather than producing GFP-tagged FV particles and examining their uptake into target cells, the authors transfected N-terminal GFP-tagged PFV Gag expression constructs. This, of course, means that they were analyzing de novo Gag expression, particle assembly and egress, and not entry processes. This raises the question, whether the identified LC8 interaction is indeed involved in FV entry. It may be that it is important for both types of transport processes; however, this needs to be carefully evaluated. Our data clearly indicate that N-terminal Gag-AFPs show a block in PFV particle egress after capsid assembly and accumulation at the centrosome, and are not suited for analysis of FV entry processes.

In contrast, particle release supported by C-terminal tagged PFV Gag proteins was similar to wild type. Differences (12 to 100-fold) in the relative infectivities of the fluorescent particles were observed, independent of the AFP tag used. However, this did not appear to correlate with differences in particle egress. Currently, the cause of these differences is unclear. However, the diminished relative infectivity observed is reminiscent of reports of other retroviral Gag-AFP fusion proteins [39,40]. Electron microscopic ultrastructural analysis of C-terminal AFP-tagged FV capsids revealed that this effect is not due to gross changes in particle morphology as observed for orthoretroviral Gag harboring a C-terminal AFP tag [41]. This suggests that pure Gag-GFP containing FV particles might have defects in disassembly, leading to the observed decrease in infectivity observed in the marker gene transfer assays. The exact cause remains to be characterized.

C-terminal GFP-tagged particles were used further to study the virus - target cell interaction. We demonstrated that selected synthetic lipids, as well as lipids extracted from susceptible target cells, do not contribute to virus adsorption or attachment as previously reported for retroviral VSV-G pseudotypes [34]. In contrast, we showed that fluorescent FV particles bind specifically to target cells. Since FVs naturally are unable to release VLPs without Env, we verified binding specificity by using a non-infectious Env-independent budding Gag mutant (PGM3). For this mutant equal amounts of Gag-driven particle release were observed independently of PFV Env coexpression. Env containing PGM3 particles showed specific binding to target cells in CLSM analysis and were more quantitative in a flow cytometric binding assay. In combination with a screen of various target cell types for susceptibility to transduction by high titer retroviral vector supernatants, this binding assay was used to characterize features of FV attachment to target cells. First, using mouse L cells, and a glycosaminoglycan synthesis deficient subclone (sog9) thereof, we found that glycosaminoglycans contribute to FV attachment and transduction. However, they are not essential for FV Env-mediated particle binding and entry and therefore seem not to represent the currently unknown but ubiquitous cellular receptor of FVs. More importantly, for the first time two cell lines were identified that are resistant to FV Env-aided gene transfer. The human erythroid precursor cell line G1E-ER4 and the zebrafish cell line Pac-2 were both resistant to transduction by FV Env-containing high-titer retroviral vectors. Transduction resistance correlated with the FV Env protein and not the nature (FV vs. HIV) of the enveloped capsid structure. However, for both cell lines, specific binding of fluorescent FV Env-containing retroviral particles to the cell surface was observed, although spinoculation had to be used for the G1E-ER4 suspension cells. Taken together these transduction and binding data suggest that FV Env-containing particles can still attach to these target cells, maybe by low-affinity scaffold interactions involving cell surface proteoglycans, for example. However, viral- and cellular lipid membrane fusion and release of the capsid into the cytoplasm of these cells are apparently blocked, potentially because this process in FV entry is dependent on a specific cellular molecule lacking in these cells. A detailed comparison of the entry processes in susceptible cells and these non-permissive target cells is required to confirm or reject this hypothesis.

## Conclusions

In summary, this study precisely describes for the first time the development of functional, fluorescent FV particles, opening up a new field in Foamy virus research. Moreover, the identification of the non-permissive G1E-ER4 and Pac-2 cells will allow further insight into various steps of the FV replication cycle, in particular concerning virus entry and potentially identification of its currently unknown ubiquitous cellular receptor.

## Methods

### Cells

The human kidney cell line 293T [42], the human fibrosarcoma cell line HT1080 [43], the mouse L, the sog9 [36] and the HeLa cell line [44] were cultivated in Dulbecco's modified Eagle's medium supplemented with 10% heat-inactivated fetal calf serum and antibiotics. HeLa cells were cultivated in phenol red free media. The zebrafish embryonic fibroblast cell line Pac2 was cultivated in Leibovitz media L15 supplemented with 20% heat-inactivated fetal bovine serum and antibiotics at 28°C [45,46]. The suspension T-cell line Jurkat [47] was cultivated in RPMI-1640 media supplemented with 10% heat-inactivated fetal calf serum and antibiotics. The immortalized, erythroid suspension cell line G1E-ER4 was cultivated in Iscove's modified Dulbecco's medium, supplemented with 15% heat-inactivated fetal calf serum, recombinant human erythropoietin (2 U/ml) and recombinant rat SCF (50 ng/ml) [48].

### Expression constructs

The original 4-plasmid PFV vector system consisting of the PFV Gag expression vector pcziPG4, the PFV Pol expression vector pcziPol, the PFV Env expression construct pczHFVenvEM002, and the enhanced green fluorescent protein (EGFP)-expressing PFV transfer vector pMD9, has been described previously [29]. In this study an expression-optimized PFV Gag construct pcoPG4 (PG) was used instead of the original pcziGag4 construct that contains the wild type PFV Gag ORF. Expression-optimization and gene synthesis was done by Geneart, Regensburg, Germany. Furthermore, a variant transfer vector puc2MD9 was used, containing a pUC19 backbone with a SV40 ori instead of the pcDNA3.1 zeo backbone of the original pMD9 vector. For some experiments the PFV transfer vector pMD11, encoding lacZ as reporter gene, was used [29]. A schematic outline of the PFV Gag constructs used in this study is shown in Fig. 1. Expression vectors for Gag fusion proteins were cloned by fusing the tag sequence (HA, EGFP, EYFP, mCerulean or mCherry), together with a flexible glycine-serine (G/S) linker in between, either N- (e.g. pcoPG4 NeGFP) or C-terminal (e.g. pcoPG4 CeGFP) to the PFV Gag ORF in pcoPG4. Further modification of pcoPG4 CeGFP by addition of an N-terminal tag comprising the c-src membrane-targeting signal (aa 1-10), a HA tag, a flexible G/S linker and an additional N-terminal Gag p68/p3 proteolytic cleavage site to the full-length PFV Gag ORF resulted in the generation of the PGM3 mutant, allowing PFV Env-independent membrane-targeting (Lindemann, unpublished data). All Gag fusion protein (FP) expression constructs were generated using standard PCR cloning techniques and mutagenesis primers and were verified by sequencing analysis. Details are available upon request. In some transduction experiments a replication-deficient lentiviral vector system was used. HIV-1 pseudotyped viruses were generated by cotransfection of the constitutively EGFP expressing lentiviral transfer vector p6NST90 (Lindemann, unpublished) and the packaging plasmids pCD/NL-BH and pczVSV-G encoding for HIV-1 Gag/Pol and the VSV-G protein (Vesicular Stomatitis Virus glycoprotein G), respectively [49,50].

### Generation of viral supernatants and analysis of transduction efficiency

FV supernatants containing recombinant viral particles were generated essentially as described previously [51,52]. Briefly, FV supernatants were produced by cotransfection of 293T cells with transfer vector (puc2MD9 or pMD11), Env- (pczHFVenvEM002), Pol - (pcziPol), and Gag packaging plasmid (pcoPG4 or PG mutants thereof as indicated) at a ratio of 4:4:4:1 using polyethyleneimine (PEI) or Polyfect transfection reagents. At 24 h posttransfection, sodium butyrate (final concentration, 10 mM) was added to the growth medium for 8 h. Subsequently, the medium was replaced, and viral supernatants were harvested an additional 16 h later. Lentiviral supernatants were generated by cotransfection of transfer vector (p6NST90), Gag/Pol packaging plasmid (pCD/NL-BH), and an Env packaging plasmid (pczVSV-G or pczPE01) at a ratio of 1:1:1 and harvested as described above.

Transductions of recombinant EGFP expressing PFV vector particles containing various PFV Gag proteins were performed by infection of 2 × 10^4 ^HT1080 cells, plated 24 h in advance in 12-well plates. For the infection 1 ml of the viral supernatant or dilutions thereof were incubated with the target cells for 4 to 6 h. The percentage of EGFP-positive cells was determined by fluorescence-activated cell sorter (FACS) analysis 72 h after infection. All transduction experiments were performed three times, and in each independent experiment the values obtained with the wild-type construct pcoPG4 were arbitrarily set to 100%. Analysis of tissue tropism of different PFV vector particles or HIV-1 vector pseudotypes was performed on various target cell lines. Adherent target cells (HT1080, HeLa, mouseL, Sog9, Pac2), which were plated one day in advance at a density of 2 × 10^4 ^cells in 12-well plates, were infected with one ml of viral cell culture supernatant or dilutions thereof. Target cells growing in suspension (Jurkat, G1E-ER4) were infected by resuspending 1 × 10^5 ^target cells in one ml of viral cell culture supernatant or dilutions thereof. Afterwards they were transferred into a 6-well plate and either incubated at 37°C, 5% CO_2 _in a humidified incubator or centrifuged for 1 h at 2000 rpm (30°C) before the incubation step. Sixteen hours later the viral cell culture supernatant was replaced for both types of target cells by fresh media and the transduction efficiency was determined by flow cytometry 72 - 96 h after infection as described above.

### Biochemical analysis of PFV particles

Biochemical analysis of purified PFV particles was essentially performed as described previously [15,53]. Briefly, the cell-free viral supernatant, generated by transient transfection of 293T cells as described above, was harvested by sterile filtration (pore size, 0.45 μm) and centrifuged at 4°C and 25,000 rpm for 3 h in an SW40 or SW28 rotor through a 20% sucrose cushion. The supernatant was discarded, and the viral pellet was gently resuspended in 50 μl phosphate-buffered saline (PBS). An equal volume of 2× sodium dodecyl sulfate (SDS) protein sample buffer (2×PPPC) was added to the samples, which were separated by SDS-polyacrylamide gel electrophoresis (PAGE) and analyzed by Western Blotting as described below. In some experiments viral particles were resuspended in a larger volume (134 μl PBS). Subsequently, 67 μl of each sample were proteolytically digested with subtilisin (0.5 mg/ml), the other part was incubated with PBS instead, for 2 h at 37°C. Digestion was terminated by addition of 2 μl phenylmethylsulfonyl fluoride (20 mg/ml) and 6×PPPC.

### Antisera, Western blot expression analysis

Western blot expression analysis of cell- and particle-associated viral proteins was performed essentially as described previously [53]. Polyclonal antisera used were specific for PFV Gag [54] or the LP of PFV Env, aa 1 to 86 [53]. The chemiluminescence signal was digitally recorded using a LAS-3000 imager.

### FACS based analysis of viral particle target cell binding

A flow cytometric assay was applied to determine and compare the capability of individual particle preparations to specifically bind to selected target cells. GFP-tagged PFV PG or PFV PGM3 derived particles of 10 ml viral supernatant, generated by transient transfection of 293T cells as described above, were harvested, concentrated by ultracentrifugation and resuspended in 100 μl PBS (phosphate-buffered saline) containing 10% inactivated fetal calf serum. HIV VSV-G pseudoparticles were generated as described previously [40]. Adherent target cells were detached with PBS-EDTA. Subsequently, 5 × 10^4 ^cells were incubated with virus or dilutions thereof in a total volume of 100 μl. After 30 min incubation on ice, cells were washed two times with FACS buffer (phosphate buffered saline, 1% inactivated fetal calf serum). For the competition experiment, untagged PFV particles or dilutions thereof were incubated 30 min on ice, washed two times with FACS buffer and subsequently incubated another 30 min with GFP-tagged PFV particles. After a fixation step with 6% formaldehyde, the cell pellets were resuspended in 150 μl FACS buffer, stored on ice, and analyzed by flow cytometry using a FACS Calibur (Becton Dickinson). The mean fluorescence of 8,000 - 10,000 events per sample was subsequently determined using the Cell Quest software package (Becton Dickinson).

### Lipid binding assays

Lipid binding assays were performed with purified PFV particles or HIV pseudoparticles, generated as described above. Synthetic lipids were obtained from Avanti Polar Lipids, Alabaster, USA. Lipid extracts were prepared from non-transfected HeLaP4 cells according to Dreyfus et al. [55]. This is a modified method according to Folch-Pi et al. with optimizations for recovering highly polar lipids like Gangliosides [56]. Briefly, cells were grown to near confluency, washed with PBS and 150 mM NaCl and were harvested by gentle scraping. 5 × 10^7 ^cells were suspended in a total volume of 450 μl of 150 mM NaCl. All following solvent extraction steps were carried out in a glass vial. 5 ml of chloroform/methanol (1:1, v/v) was added. To facilitate extraction of lipids, the suspension was sonicated for 30 min in a bath sonicator interrupted by 2 × 5 min of vortexing. Insoluble material was pelleted by centrifugation at 3200 × g for 10 min and subsequently subjected to 2 further extraction steps. Three ml of chloroform/methanol (1:1) or chloroform/methanol/H_2_O (48:35:10, v/v), respectively, were added and extraction and centrifugation were performed as before. All 3 supernatants were collected, dried under nitrogen and redissolved in 1.5 ml chloroform/methanol/H_2_O (40:20:3, v/v).

For the lipid spot binding assay small spots of lipid solutions of about 25 mg/ml in organic solvent were dried on cover slips. Remaining traces of solvent were removed by applying vacuum for at least 1 h. The spots were covered by PBS, which contained fluorescent viral particles. The concentration of viral particles had been measured by fluorescence correlation spectroscopy (FCS) before and adjusted accordingly. Confocal laser scanning images of the surface of the lipid spots were obtained on a Zeiss LSM 510 and evaluated by ImageJ and custom made Perl scripts. In short, particles were detected and counted in 3 subsequent images after smoothing and intensity thresholding. Diffusing particles that appear blurred due to slow scanning were discarded based on their low circularity (sqrt(area)/perimeter < 0.21). For a quality control of the lipid extract the binding of cholera toxin subunit B and Annexin V was observed in PBS or PBS containing 2 mM CaCl_2_, respectively. The toxin and Annexin V both bound to the spotted lipid extract but not to synthetic DOPC and only Annexin V bound to DOPS. This shows that both charged lipids as phosphatidyl serine (detected by Annexin V) and highly polar glycolipids like GM1 (detected by cholera toxin) were extracted.

Giant unilamellar vesicles (GUVs) were produced by electroformation [57]. In short, lipids were dried on opposed indium tin oxide (ITO) coated coverslips and the resulting chamber was filled with 465 mM sucrose/2 mM EDTA. An alternating voltage of 2 V/10 Hz was applied for 2 h in order for the GUVs to form. These vesicles were sedimented in double the volume of 1.5 × PBS. Virus particles were added before observation of binding in confocal microscopy.

### Combined Atomic Force Microscopy (AFM) and Confocal Fluorescence Microscopy

PFV particles were harvested as described above and additionally fixed by addition of PFA (paraformaldehyde) at a final concentration of 2% to the cell culture supernatant and to the 20% sucrose cushion prior to ultracentrifugation.

AFM and fluorescence imaging were performed at room temperature using the same experimental apparatus. It consisted of a NanoWizard AFM (JPK Instruments, Berlin, Germany) mounted on a Laser scanning microscope (LSM) 510 Meta (Zeiss, Jena, Germany). For AFM imaging, uncoated silicon cantilevers (MikroMasch, Spain) with typical spring constant of 0.3 N/m (manufacturer specified) were used in intermittent contact mode. The cantilever oscillation was tuned to a frequency of ~5 kHz, with a maximum amplitude set to 0.1- 0.15 V (5-7.5 nm). The scan rate was set to 0.7- 1 Hz. The height, error, and phase-shift signals were collected simultaneously in both trace and retrace directions. Images were line-fitted as required. Isolated scan lines were occasionally removed. For confocal fluorescence microscopy, the excitation light of an Argon laser at 488 nm was reflected by a dichroic mirror (HFT 490) and focused onto the sample by a Zeiss C-Apochromat 40×, NA = 1.2 UV-VIS-IR water immersion objective. Fluorescence signal was then recollected by the same objective and, after passing through a 530/30 bandpass filter, measured by a photomultiplier (PMT). The confocal geometry was ensured by a 70 nm pinhole in front of the PMT.

Precise spatial alignment of fluorescence imaging and AFM was achieved using 0.1 μm size fluorescent carboxyl-modified polystyrene beads (F-8888, Molecular Probes, Eugene, OR) as "topographical landmarks". Viral particles and fluorescent beads were mixed in phosphate buffer (1 mM KCl, 0.5 mM KH_2_PO_4_, 2.7 mM Na_2_HPO_4_, 50 mM NaCl, pH 7.2) and deposited on a thin fresh cleaved mica sheet previously treated for 10 minutes with a 0.1 mg/ml poly-D-lysine (P7280, Sigma) solution. After ca. 20 minutes, the sample was rinsed with the same phosphate buffer and then ready for imaging.

### CLSM analysis of PFV binding to host cells

Host cells were seeded at a density of 1.5 × 10^4 ^cells/well into 8 well chamber slides. After 24 h cells were cooled down and incubated on ice with fluorescent PFV particle preparations for 30 min. Subsequently cells were washed with cold PBS and either fixed with 3% PFA or incubated an additional 30 min at 37°C before fixation. After fixation the cell nuclei were stained with DAPI for 5 min. Finally the cells were covered with 50% glycerol (in water). Confocal laser scanning images were obtained on a Zeiss LSM 510 and evaluated by ImageJ. The excitation light of an Argon laser at 488 nm or a Diode laser at 405 nm was reflected by a dichroic mirror (HFT 405/488/561) and focused onto the sample by a Zeiss Apochromat 63×, NA = 1.4 oil immersion objective. Fluorescence signal was then recollected by the same objective, splitted by a dichroic mirror (NFT 490) and after passing through either a 520/30 or 420/60 bandpass filter, measured by a photomultiplier (PMT). The confocal geometry was ensured by a 1 Airy Unit pinhole in front of the PMT.

### Electron microscopy analysis

At 48 h post transfection, the 293T cells were harvested and processed for electron microscopy analysis as described previously [58].

## Competing interests

The authors declare that they have no competing interests.

## Authors' contributions

DLü, ASt, ASw, EM, JR and AG carried out the basic characterization of some to the constructs. SC and HK performed all biophysical experiments using AFM and LSM microscopy in cooperation with KS. NS and HH contributed in the cellular susceptibility screening experiments. HWZ performed the electron microscopy analysis. PS and DLi made substantial contributions to conception and experimental design of the study. Furthermore they were mainly involved in interpretation of data and drafting the manuscript. KS contributed to the experimental design, performed all main experiments on her own, coordinated and participated in collaborative experiments, and was involved in drafting the manuscript. All authors read and approved the final manuscript.
